# Improving facilitation of ECT treatment for patients in an acute medical hospital

**DOI:** 10.1192/bjo.2021.162

**Published:** 2021-06-18

**Authors:** Vatsala Mishra, Chun Chiang Sin Fai Lam, Marilia Calcia, Isabel McMullen

**Affiliations:** Mental Health Liaison Team, King's College Hospital, South London and Maudsley NHS Foundation Trust

## Abstract

**Aims:**

A Quality Improvement Project aiming to streamline facilitation of electroconvulsive therapy (ECT) treatment for psychiatric patients at a general acute hospital and reduce cancellation rates via the use of a checklist.

ECT treatment is an essential aspect of psychiatric care for patients with severe depression or treatment-resistant psychosis. Facilitation of ECT treatment is an uncommon task for liaison psychiatry and the medical and nursing teams responsible for patients’ medical care. Between August-October 2019, this liaison psychiatry team had 3 patients undergoing ECT treatment a total of 13 times, with treatment being cancelled on 4 occasions. After engagement with stakeholders from the acute medical teams, the liaison team and the ECT suite team, key areas requiring intervention were identified to help reduce the rates of cancellation. Areas identified included a lack of ownership on the logistic and operational aspects of ECT amongst staff, a lack of knowledge of what the process involved and a lack of confidence in managing said patients. Difficulties in communication between teams and accurate documentation may contribute to errors and cancellation of ECT sessions, which in turn would delay treatment and impact on patient safety and clinical outcomes.

**Method:**

The first author, a Foundation Year 1 doctor, developed a 10-point checklist to be referred to when arranging ECT for patients, to ensure errors were not made which could lead to missed treatment and delayed recovery. The tasks and responsibilities of each key member of the team were clearly identified. This checklist was included in all ECT patients’ files and teaching was provided to staff involved. Feedback was obtained from staff involved regarding the clarity of information and their confidence in managing such cases.

**Result:**

In the month following initial intervention the liaison psychiatry team organised 12 ECT sessions. The checklist was pasted into notes the day before each ECT session and 0 sessions were missed for avoidable reasons. Feedback from staff showed all teams felt more confident co-ordinating ECT treatment as a result of the checklist.

**Conclusion:**

Creating a 10-point checklist for the facilitation of ECT treatment in patients at a medical hospital was beneficial in reducing avoidable errors from 16% to 0%. The liaison psychiatrists, medical doctors, and nurses involved reported greater confidence in managing patients undergoing ECT and described the checklist as enhancing the feeling of teamwork and communication within the multi-disciplinary team, and felt it had improved patient safety and clinical outcomes.

